# Supplementary material for: Radiofrequency Irradiation Attenuates High-Mobility Group Box 1 and Toll-like Receptor Activation in Ultraviolet B–Induced Skin Inflammation
Source: Molecules. 2021 Feb 28;26(5):1297. doi: 10.3390/molecules26051297 (PMC7957621; doi:10.3390/molecules26051297)
Supplement: Supplementary file 1 [file molecules-26-01297-s001.pdf]

Supplementary Tables

Table S1. List of primer for Quantitative polymerase chain reaction (qRT-PCR)

Gene		Primers
β-actin	Forward	5'-ACA AAG CTG TTC AGT GTC TCC A-3′
	Reverse	5'-CTC CGT TTC CAG AAT ACA CAC A-3′
Cyclin D1	Forward	5'-AAT GGA ACT GCT TCT GGT GAA C-3′
	Reverse	5'-AGG AAG TGT TCG ATG AAA TCG T-3′
p21	Forward	5'-GAC AAG AGG CCC AGT ACT TCC-3′
	Reverse	5'-CTC AGA CAC CAG AGT GCA AGA C-3′
p53	Forward	5'-TGT CAT CTT TTG TCC CTT CTC A-3′
	Reverse	5'-CAC ATA ACA GAC TTG GCT GTC C-3′
Bcl2	Forward	5'-ATG CGA CCT CTG TTT GAT TTC T-3′
	Reverse	5'-AGG TAT GCA CCC AGA GTG ATG-3′
TNF-α	Forward	5'-CAT GGA TCT CAA AGA CAA CCA A-3′
	Reverse	5'-CCT TGA AGA GAA CCT GGG AGT A-3′
IL-1ß	Forward	5'-ACC AAG CAA CGA CAA AAT ACC T-3′
	Reverse	5'-CCG TCT TTC ATT ACA CAG GAC A-3′
CCL5	Forward	5'-CTC CAA TCT TGC AGT CGT GTT-3′
	Reverse	5'-CTT GAA CCC ACT TCT TCT CTG G-3′
CCL20	Forward	5'-CAG AAG CAA GCA ACT ACG ACT G-3′
	Reverse	5'-TGT GAA ACC CAC AAT AGC TCT G-3′
IL17	Forward	5'-AAG GAC TTC CTC CAG AAT GTG A-3′
	Reverse	5'-GAA CGG TTG AGG TAG TCT GAG G-3′
VEGF	Forward	5'-GTT TAA ATC CTG GAG CGT TCA C-3′
	Reverse	5'-TTT GCA GGA ACA TTT ACA CGT C-3′
VEGFR2	Forward	5'-GAA ACA GAA TTT CCT GGG ACA G-3′
	Reverse	5'-TTG ATC TTT GCC TCA CAG AAG A-3′
HSP27	Forward	5'-ATG ATG GCA TCC TTA ACT TGG A-3′
	Reverse	5'-CAG GAA GCA GGG AGA TGT AGA C-3′
FAK	Forward	5'-CCA CCC TCT ACA GCC TTA TGA C-3′
	Reverse	5'-TTG TGC TGA GCT GAG CTT TTA G-3′

Supplementary Figures

Figure S1. Inhibitory effects of RF irradiation on Iba1 activation in UVB-radiated mouse skin *in vivo*

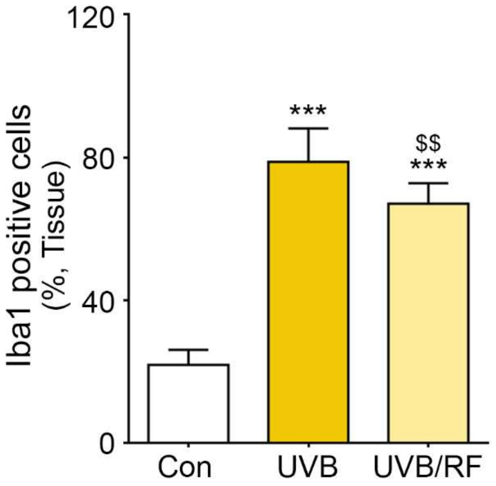

The graphs showing Iba1 positive cell number of representative Immunohistochemistry result. ***, $P < 0.001$ vs. CON group; $$, $P < 0.01$ vs. UVB group. Results are presented as means ± SD. CON, sham control; UVB, ultraviolet B; RF, radiofrequency

Figure S2. Inhibitory effects of RF irradiation on TLR2 and TLR4 expression in activated macrophage *in vivo*

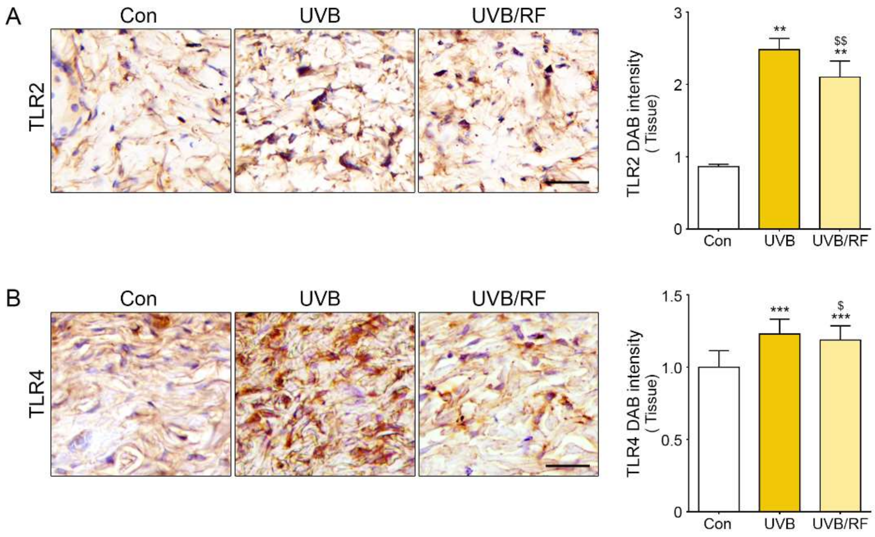

(A) Immunohistochemistry result showing TLR2 expression in the skin dermis of UVB-radiated mouse and graph showing intensity of TLR2 from representative results. (B) Immunohistochemistry result showing TLR4 expression in the skin dermis of UVB-radiated mouse and graph showing intensity of TLR4 from representative results. Scale bar =100 μm. Magnification, ×40. **, P < 0.01 and ***, P < 0.001 vs. Con group; $, P < 0.05 and $$, P < 0.01 vs. UVB group. Results are presented as means ± SD. Con, sham control; RF, radiofrequency; TLR2, Toll-like receptor 2; TLR4, Toll-like receptor 4; UVB, ultraviolet B

Figure S3. Inhibitory effects of RF irradiation on keratinocyte proliferation, apoptosis regulator and pro-inflammatory factor expression through macrophage modulation *in vitro*

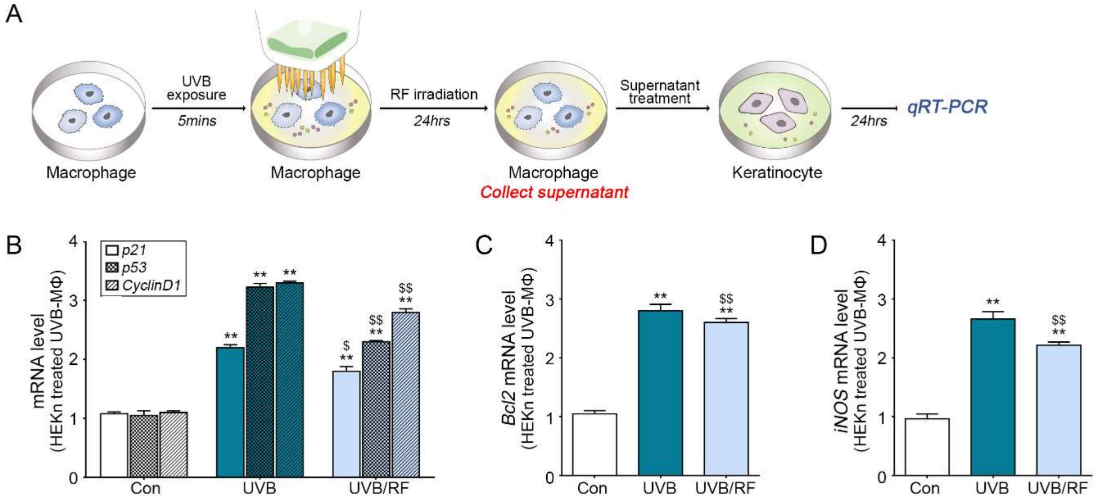

(A) Diagram of an in vitro model to examine the effect of microneedling RF irradiation to UVB-radiated macrophages on the inflammatory response in keratinocytes. After exposing macrophages to UVB, UVB/RF, the cell culture supernatants were used to treat keratinocyte for 24 hours. (B) The graphs showing mRNA levels of p21, p53, Cyclin D1, a cell proliferation marker, and (C) the graph showing Bcl2 mRNA level, an apoptosis regulator gene, was measured in keratinocytes affected UVB-radiated macrophages. (D) The graphs showing mRNA levels of iNOS, a pro-inflammatory factor, was measured in keratinocytes affected UVB-radiated macrophages. **, $P < 0.01$ vs. Con group; $, $P < 0.05$ and $$, $P < 0.01$ vs. UVB group. Results are presented as means ± SD. RF, radiofrequency; UVB, ultraviolet B

Figure S4. Inhibitory effects of RF irradiation on inflammatory mediator expression *in vivo*

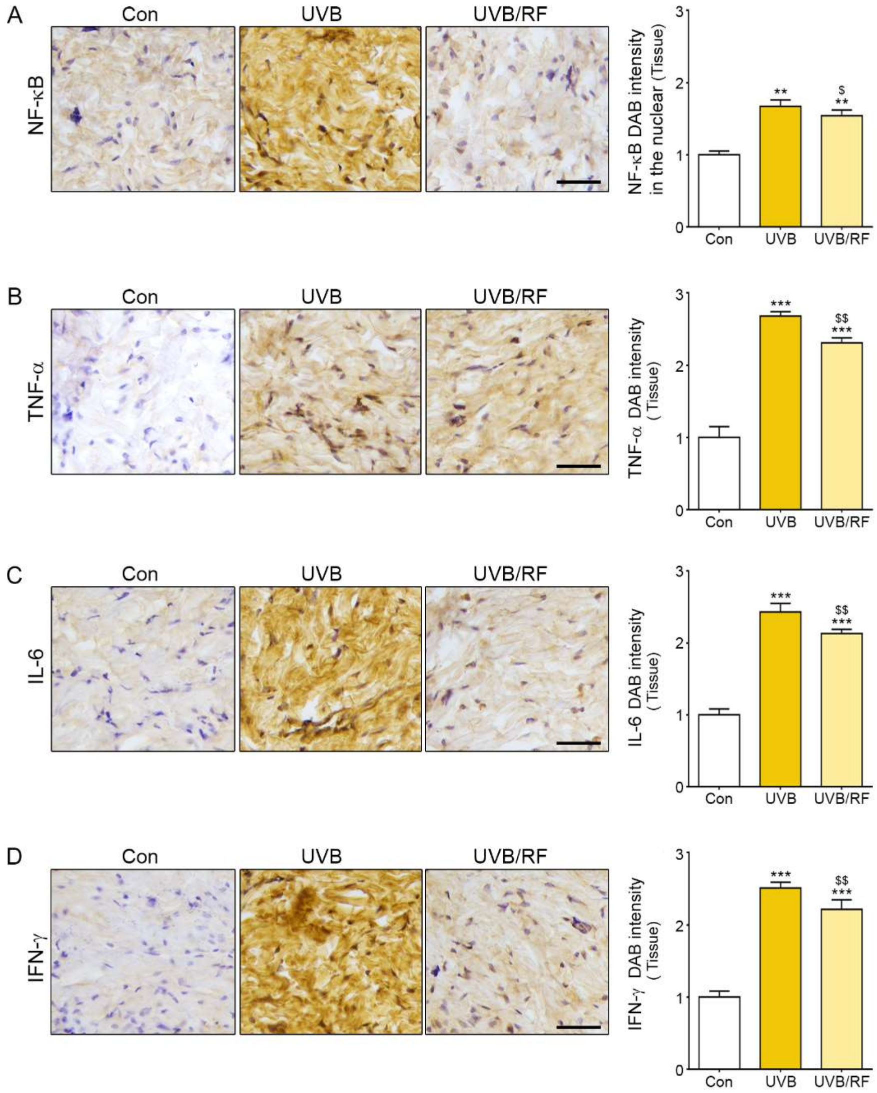

(A) Immunohistochemistry result showing NF-κB expression in the skin dermis of UVB-radiated mouse and graph showing intensity of NF-κB from representative results. (B) Immunohistochemistry result showing TNF-α expression in the skin dermis of UVB-radiated mouse and graph showing intensity of TNF-α from representative results. (C) Immunohistochemistry result showing IL-6 expression in the skin dermis of UVB-radiated mouse and graph showing intensity of IL-6 from representative results. (D) Immunohistochemistry result showing IFN-γ expression in the skin dermis of UVB-radiated mouse and graph showing intensity of IFN-γ from representative results. Scale bar =100 μm. Magnification, ×40. **, $P < 0.01$ and ***, $P < 0.001$ vs. Con group; $, $P < 0.05$ and $$, $P < 0.01$ vs. UVB group. Results are presented as means ± SD. Con, sham control; RF, radiofrequency; NF-κB, nuclear factor kappa-light-chain-enhancer of activated B cells; TNF-α, tumor necrosis factor alpha; IL-6, interleukin-6; IFN-γ, interferon-gamma; UVB, ultraviolet B
